# An audit of breast cancer in patients 40 years and younger in two Johannesburg academic hospitals

**DOI:** 10.4102/sajr.v28i1.2772

**Published:** 2024-03-28

**Authors:** Nthabiseng Chaane, Marianne Kuehnast, Grace Rubin

**Affiliations:** 1 Department of Radiology, Faculty of Health Sciences, University of the Witwatersrand, Johannesburg, South Africa

**Keywords:** breast cancer, age, 40 years, young cancer, Charlotte Maxeke Johannesburg Academic Hospital, Chris Hani Baragwanath Academic Hospital

## Abstract

**Background:**

Breast cancer is the most common cancer in females, usually diagnosed after the age of 50 years. There is a perceived increase in breast cancer cases in young women in two public sector Johannesburg academic hospitals; however, there is a shortage of data to confirm this.

**Objectives:**

This study aimed to assess data on breast cancer in young patients and determine any increase in the number of cases in patients 40 years and younger.

**Method:**

A retrospective analysis of radiology and histopathology reports of patients 40 years and younger, seen at the radiology departments of two Johannesburg academic hospitals, was performed over a 5-year period. The frequency, histology and immunohistochemical results of breast cancer diagnoses were determined in patients with a Breast Imaging – Reporting and Data System (BI-RADS) classification of 4 or above.

**Results:**

Breast cancer was diagnosed in 73% of the total eligible 469 patients. The mean patient age was 34.35 years. Invasive ductal carcinoma was diagnosed in 83% (*n* = 283) of patients classified as BI-RADS 5 on imaging. Luminal A and B subtypes were the most common. The highest number of patients (*n* = 142) were seen in 2016 of which 92 had breast cancer.

**Conclusion:**

In this very specific sample set, there was a lower number of breast cancer diagnoses in 2015 and then an increase of breast cancer diagnoses in young patients from 2016 to 2018.

**Contribution:**

Earlier breast cancer detection benefits the patient, their families and their reproductive ability. Knowledge of breast cancers in young patients can increase awareness, leading to effective, early diagnoses.

## Introduction

Breast cancer is considered one of the leading causes of death in females, affecting one in four women globally, with over 2 million cases according to Globocan 2020.^1^ The National Cancer Registry showed breast cancer was the leading invasive cancer in females in South Africa in 2019.^2^ Shah et al. reported that breast cancer was the most frequently diagnosed cancer between the ages of 55 years and 64 years with a median age of death of 68 years.^3^ The American Cancer Society shows an increase in invasive breast cancers across all ages from ‘21 to 50+’ years of age.^4^ The incidence rates of breast cancer have also increased dramatically in Northern and Southern Africa from ‘23.3 per 100 000 to 48.9 per 100 000’ from 2002 to 2018 while the incidence rate has stayed constant in Eastern, Central and Western Africa.^5^

Breast screening programmes around the world focus on average risk females over the age of 40 years, who present annually for imaging.^4^ The current clinical and breast imaging guidelines for South Africa focus on women aged 40 years and older, with the aim of early detection, diagnosis and treatment.^6^ In South Africa, mammography (MMG), ultrasonography (USG) and MRI are available for the diagnosis of breast cancer in most tertiary and quaternary hospitals. Imaging is also used to investigate clinical signs and symptoms of breast pathology such as palpable lumps and mastalgia.^7^

No screening programmes focus on or include patients younger than 40 years because of the stratified risk of breast cancer according to age.^8^ Patients considered ‘young’ (under the age of 40 years) are usually referred to breast clinics for imaging if they present to a healthcare provider with a breast-related symptom. The South African guidelines suggest referral to a breast clinic for patients over 25 years with the following symptoms: palpable or fixed lump, axillary lymph nodes, skin tethering, nipple retraction changes or nipple discharge.^6^

Azim et al. reported that breast cancer is predominantly diagnosed in patients below 40 years in 5% – 7% of patients in developed countries and close to 20% in developing countries.^9^ There is a lack of data on breast cancer diagnoses in young patients in South Africa. Multiple studies have attempted to determine the risk factors associated with breast cancer in patients under the age of 40 years. Anders et al. mentioned a positive family history of breast cancer with 50% of females under the age of 30 years, having genetic mutations.^10^ Other risk factors included breast density, contraceptive use, early menarche, obesity and previous radiation, but these factors do not differ from their older counterparts.^10^

Studies have reported that breast cancers in younger patients are more aggressive with poorer outcomes and require more aggressive therapy.^8,9,11,12,13^ An American study reported that in patients under the age of 40 years, breast cancer is the leading cause of cancer-related deaths and that cancer found in these young women was likely to be ‘larger, lymph node-positive and have an increased risk of recurrence’.^13^ Patients aged less than 40 years were shown to be 30% more likely to die of their breast cancer than their middle-aged counterparts after adjustment for treatment, stage, tumour grade and molecular subtype.^14^ Breast cancer diagnosed at this age has been considered a poor prognostic factor because of the risk of recurrence.^14^

A study by Eugênio et al. found that the MMG features of breast cancer in patients younger than 40 years were suspicious masses, asymmetric breast tissue and suspicious calcifications.^15^ The same study found that on USG, hypoechoic masses with suspicious shapes and margins made up 74.8% of their cancers.^15^ Multiple studies confirm that the above features are highly suspicious for malignant lesions with a similar Breast Imaging – Reporting and Data System (BI-RADS) positive predictive value (PPV) in young and old patients.^16,17^ Breast cancer diagnosis differs only slightly between young and old patients in that young patients are more likely to have only an USG and not a MMG in their workup.

Multiple reasons for delayed diagnosis of breast cancer in young patients have been formulated, including but not limited to poor education, limited health-seeking behaviour and limited access to screening programmes.^15^ Difficulty in clinical and radiological diagnosis in patients who are not the target group for screening programmes is another reason for delayed diagnosis.^12^ The increased breast density in this age group is a factor that directly affects MMG findings and diagnosis.^18^ Dense breast tissue is replaced by fat as age increases, and the Cancer Association of South Africa (CANSA) found that 44% of women in their early 40s have heterogeneously dense breasts, while 14% have extremely dense breasts, as per the BIRADS classification.^2^ Patients with dense breasts are also found to have an increased risk of breast cancer; their cancers are more difficult to detect and they have a 17 times higher risk of interval cancers.^18^

The BI-RADS classification is used in all our institutions and has been shown to improve the quality of patient care by standardising reports.^19^ The use of BI-RADS is associated with improved prognostication and diagnosis of cancer as well as guiding the necessary further follow-up imaging. Many studies have found differing PPV of BI-RADS 0, but Timmers et al. found that in women aged 49–75 years, the BI-RADS PPV increased with age with little interobserver variation for the BI-RADS 5 classification.^19^

The staging, grading and histological findings will determine treatment for breast cancer and a cohort study in patients under the age of 45 years found that tumours were larger, of higher grade, more likely to involve lymph nodes, had lower oestrogen receptor (ER) positivity, had higher human epidermal receptor 2 (HER2) overexpression and had poorer overall survival.^11,14^ A 2016 study found that not only was there a similar outcome for young and old patients with HER2-positive and triple-negative disease but also that age was an independent prognostic factor in patients with luminal breast cancers.^14^

Although there is limited available data on the overall effect of breast cancer, there are assumed economic, social and reproductive effects. Patients in this age group are part of the workforce and in their reproductive years, so the effects can be vast. Nulliparity is a known risk factor for breast cancer. Interestingly, Azim et al. found that pregnancy was protective against ER-positive tumours and breastfeeding was protective against triple-negative cancers, even in high-risk populations.^9^

The principal objective of this study was to assess the number of cases of breast cancer in patients younger than 40 years from 2014 to 2018 and determine whether there was any increase over the 5 years. The second objective was to document the patient’s presenting symptoms, the given BI-RADS for each breast, the size of the lesion if present, the presence of axillary lymph nodes and the liver ultrasound findings. The third objective was to obtain the histological types and molecular subtypes seen in this sample of patients.

## Material and methods

This retrospective audit included female patients, aged 40 years and younger, imaged from 01 January 2014 to 31 December 2018, and classified as BI-RADS 4 or above. Age was placed as a filter while searching on the Picture Archiving and Communication System (PACS). Excluded were male patients, female patients 41 years and older, and patients classified as BI-RADS 3 or below. The study population consisted of patients seen or referred to Charlotte Maxeke Johannesburg Academic Hospital (CMJAH) and Chris Hani Baragwanath Academic Hospital (CHBH) for a breast complaint. Convenience sampling was used as only patients on the PACS system were part of the study.

Patients younger than 40 years are usually assessed initially with USG. Ultrasound and/or MMG reports were accessed via the PACS at both CMJAH and CHBH over the 5 years. Details of age, symptoms, lateralisation, imaging, size and final BI-RADS were collected. Once all the data were collated, any repeat patients were removed.

The National Health Laboratory Service (NHLS) online database was accessed for histology and molecular subtyping. The type, subtype and hormone status were taken as described in the report. The immunohistochemical status was grouped as is commonly accepted: luminal A, luminal B, HER2-enriched and triple-negative. Any patients with no histology documented were removed from the sample. All non-breast cancer diagnoses were also captured as there was an initial suspicion of cancer.

Data were collected using a data collection sheet and entered anonymously into a Microsoft Excel spreadsheet. Patient’s presenting complaint, BI-RADS category and histology reference number were collected. In both institutions, the standard practice is to perform a liver sonar on all patients who have suspected breast cancer; these data were also collected from the radiology reports.

### Data analysis and statistics

Data analysis and descriptive analytics were performed using IBM^®^ SPSS^®^ Statistics 28.0.0. Mean, medians and percentages were calculated for numerical data, and frequencies and percentages for categorical data. Descriptive statistics included the age of the patient, the tumour size, presenting complaints, histopathology, molecular subtype, axillary node and liver findings.

### Ethical considerations

Ethical clearance to conduct this study was obtained from the University of the Witwatersrand, Human Research Ethics Committee (No. M190965).

## Results

**FIGURE 1:**
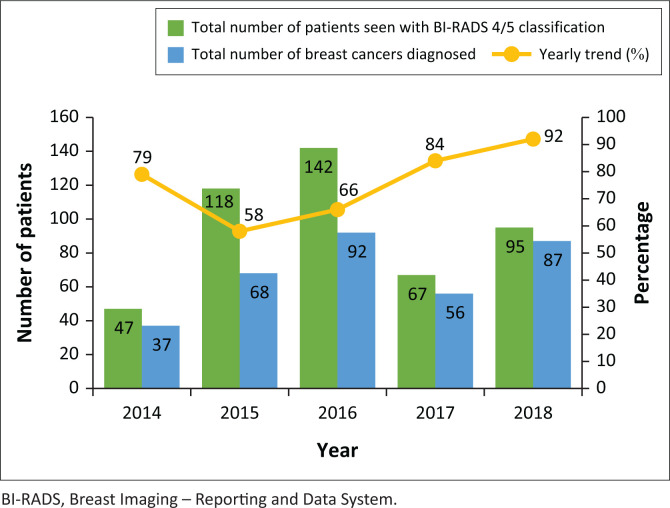
Number of patients seen with suspected breast cancer in patients 40 years and younger over the 5-year period at the two hospitals.

The data were collected from 469 patients who fulfilled the age criteria and were seen at both hospitals over the 5-year period. A total of 73% (*n* = 342) of these patients were diagnosed with breast cancer. Benign or normal diagnoses were made in 12% (*n* = 56) of the patients, while 7% (34) of the patients had high-risk lesions or another type of cancer. Histological data were missing from 37 patients who fulfilled the age criteria. The highest number of patients seen in both hospitals was 142 patients in 2016, when the highest number of breast cancers was diagnosed. Figure 1 shows the trend of breast cancer cases in our study along with the total number of patients seen yearly; from 2016 there was an increase in the number of breast cancers diagnosed.

### Patient demographics

**FIGURE 2:**
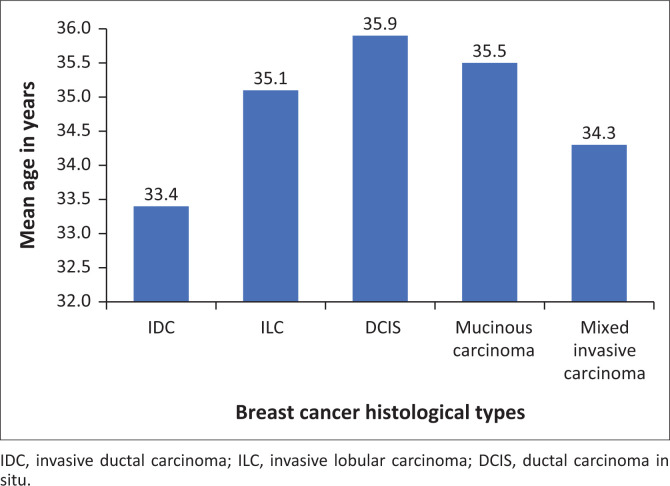
Mean age of patients by breast cancer histological type.

The age range was 15–40 years with a mean of 34.35 years and a standard deviation of 4.18 years. Only 30 patients were 40 years old at the time of the data collection. The two youngest patients were a 15-year-old diagnosed with *pseudoangiomatous stromal hyperplasia* (PASH) and a 16-year-old diagnosed with rhabdomyosarcoma. One patient aged 29 years was diagnosed with Castleman disease. Invasive ductal carcinoma (IDC) and mixed invasive carcinoma had the lowest mean ages of 33.4 years and 34.3 years, respectively. Figure 2 demonstrates the mean age of the varying histological subtypes of breast cancer.

### Presenting symptoms

The majority of patients presented with a unilateral breast mass and 88% (261 of 365) were diagnosed with IDC. The next most common cancer was ductal carcinoma in situ (DCIS), presenting with a unilateral breast mass. Bilateral breast cancers and lymphomas were the most common diagnoses in patients presenting with bilateral breast masses. Table 1 is a summary of presenting complaints by histological diagnosis and other variables. Bilateral breast cancer was found in 33% (*n* = 7) of patients who presented with bilateral breast masses. Interestingly, one patient presented with DCIS in one breast and IDC in the other breast. Two patients (10%) had unilateral breast cancer and a fibroadenoma on the contralateral side. Two patients, who presented with bilateral breast masses, only had one side biopsied.

**TABLE 1:** Main findings by cancer type.

Variables	Variable types	IDC			ILC			Mixed invasive carcinoma			Mucinous carcinoma			DCIS			Other			Total
		*n*	%	Mean	*n*	%	Mean	*n*	%	Mean	*n*	%	Mean	*n*	%	Mean	*n*	%	Mean	
Age (years)	-	-	-	33.4	-	-	35.1	-	-	34.3	-	-	35.5	-	-	35.9	-	-	-	-
Presenting complaint	Unilateral mass	261	72	-	12	3	-	1	0.3	-	1	0.3	-	20	5	-	70	19	-	365
	Bilateral mass	12	80	-	1	7	-	0	0	-	2	13	-	0	0	-	10	-	-	25
	Skin changes	5	100	-	0	0	-	0	0	-	0	0	-	0	0	-	-	-	-	5
	Nipple retraction	3	100	-	0	0	-	0	0	-	0	0	-	0	0	-	-	-	-	3
	Nipple discharge	4	80	-	0	0	-	0	0	-	0	0	-	1	20	-	-	-	-	5
Biopsy type	US-guided core needle biopsy	287	71	-	13	3	-	5	1	-	5	1	-	16	4	-	79	20	-	405
	Stereotactic biopsy	5	50	-	0	0	-	0	0	-	0	0	-	5	50	-	0	0	-	10
	FNA	3	43	-	0	0	-	3	43	-	0	0	-	0	0	-	1	14	-	7
BI-RADS	4	42	38	-	2	2	-	2	2	-	2	2	-	7	6	-	56	50	-	111
	5	254	79	-	11	3	-	3	1	-	2	1	-	17	5	-	34	11	-	321
Molecular subtype	Luminal A	130	90	-	2	1	-	3	2	-	3	2	-	7	5	-	-	-	-	145
	Luminal B	84	84	-	5	5	-	0	0	-	3	3	-	8	8	-	-	-	-	100
	HER2-enriched	27	87	-	0	0	-	2	6	-	0	0	-	1	3	-	-	-	-	31
	Triple-negative	34	87	-	1	3	-	2	5	-	0	0	-	2	5	-	-	-	-	39
Metastases	Axillary lymph nodes	57	89	-	3	5	-	3	5	-	0	0	-	1	2	-	-	-	-	64
	Liver lesions	3	75	-	0	0	-	0	0	-	1	25	-	0	0	-	-	-	-	4

IDC, invasive ductal carcinoma; ILC, invasive lobular carcinoma; HER2, human epidermal receptor 2; DCIS, ductal carcinoma in situ.

### Imaging and cytology or histology assessment

Ultrasound-guided core biopsies were the most performed biopsy technique in this study (*n* = 405). Table 1 details the biopsy type by histological diagnosis. The common histological subtypes in descending order were IDC, DCIS, invasive lobular carcinoma (ILC) and at much lower rates, mixed invasive carcinoma and mucinous carcinoma. Interestingly, of the 10 stereotactic biopsies performed, 5 revealed DCIS and the other 5 were IDC. The US-guided biopsies also revealed lymphoma, abscesses and phyllodes tumours as non-breast cancer diagnoses.

The lesions were measured in centimetres, ranging from less than 1 cm to 9 cm in diameter. A measurement of 2 cm – 3 cm was the most frequent, with few patients having lesions measuring more than 5 cm. Figure 4 shows reported lesion sizes. There was no lesion size in the majority of reports.

#### Breast Imaging – Reporting and Data System Classification

**FIGURE 3:**
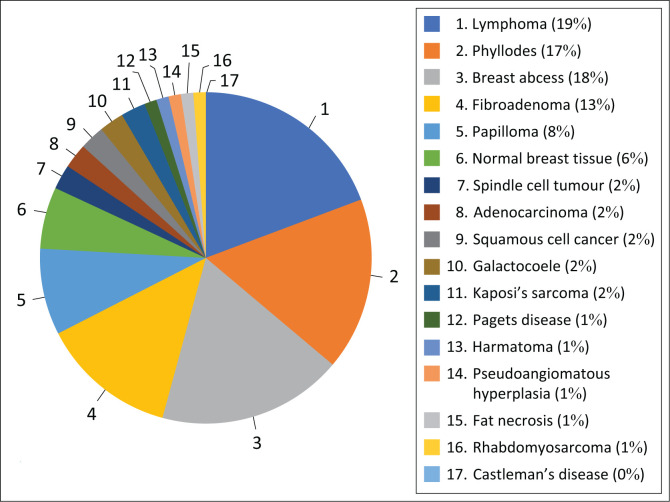
A pie-chart of the non-breast cancer diagnoses in the study population.

Breast cancer was diagnosed in 83% (287/342) of patients classified as BI-RADS 5 and 16% (55/342) classified as BI-RADS 4. Invasive ductal carcinoma was found in 86% (*n* = 296) of all patients classified as having a BI-RADS 4/5 lesion. DCIS was diagnosed in 7% (*n* = 24) of patients classified as having a BI-RADS 4/5 lesion. This study had a PPV of 89% for BI-RADS 5 lesions and a PPV of 50% for BI-RADS 4 lesions. All BI-RADS 1 classifications correlated to the unaffected breast. Of the 19% (90/469) patients who were not diagnosed with breast cancer, 34 were given a BI-RADS 5 classification and 56 were given BI-RADS 4. Figure 3 shows the number and diagnoses seen in patients not diagnosed with breast cancer.

**FIGURE 4:**
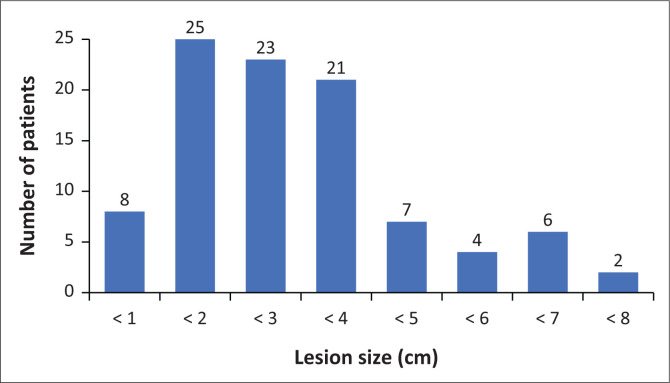
Lesion sizes in centimetres (cm) taken from the radiological report.

#### Molecular subtypes

Molecular subtype data were available in 315 patients of which luminal A and B subtypes were the most common (Table 1). Patients with triple-negative subtype tended to be older, with a mean age of 35.7 years, while HER2-enriched, luminal A and B subtypes were found in patients with a mean age of 33.6, 33.8 and 33.5 years, respectively.

#### Axillary nodes and liver metastases

Data about axillary lymph nodes and liver metastases were lacking in most reports. No suspicious axillary lymph nodes were reported in 67% (290/432) of patients, 13% (56/432) had one suspicious node and 8% (36/432) had multiple pathological nodes (Table 1). Patients with IDC had the highest number of associated axillary lymph nodes, with 89% (57/64) patients noted to have lymph nodes on initial imaging. The majority of patients with multiple (> 4) lymph nodes were diagnosed with lymphoma, and one patient was diagnosed with Castleman disease. In terms of hepatic metastases, only 1% (*n* = 4) of the study population had liver lesions on screening USG.

## Discussion

During the 5 years reviewed, there was initially a decrease in the number of breast cancer cases in 2015 despite an increase in the number of patients seen, when compared to 2014. In 2016, there was an increase in the number of breast cancers diagnosed, which continued in 2017 and 2018. The highest number of patients 40 years and below was seen in 2016, which correlates with the most cancer diagnoses. This increased trend in breast cancer diagnosis may have been because of many factors including, but not limited to, improved record keeping, more patients seen overall in each institution and more external referrals.

Statistics from the United Kingdom (UK) show a slow and steady increase in the number of cases diagnosed from 2002 to 2018^20^ while the United States has recorded an increase in cases of breast cancer in female patients younger than 40 years since 2004, with the prevalence of invasive breast cancer increasing more than other breast cancers.^16^ Johnson et al. reported that European studies found a 1.2% per year increase in breast cancer incidence and reported that breast cancer incidence in young patients is similar in developed and developing countries.^17^ McAree et al. state that the UK reports breast cancer as the most commonly diagnosed female cancer between 35 years and 39 years.^8^ Dodelzon et al. reported that despite no statistically significant increase in incidence in 10 years, an increase in invasive and later stage cancers was observed.^21^ In the face of increasing cases as seen in this study from 2016 onwards in a resource-limited, developing country like South Africa, early detection can help reduce the load on the health system as well as decrease the burden on affected patients and their families.

In this study, 86% of patients presented with a unilateral breast mass as the most common presenting complaint, which was suspicious enough to warrant further imaging and biopsy. McAree et al. found that their study cohort presented directly to the clinic with a symptom, of which 89.5% had a palpable lump.^8^ A Dutch study showed that the incidence of breast cancer in patients who presented with a palpable mass was 8%.^22^ The American Cancer Association found that almost 80% of breast cancers in young patients are found by the patients themselves.^4^

Masses (unilateral more than bilateral), nipple discharge, skin changes and axillary masses were common presenting complaints in this study. These findings along with mastalgia are common in both benign and malignant diseases.^23^ This study reminded us that axillary lymphadenopathy can also be the manifestation of other diseases like lymphoma, Castleman disease and Kaposi disease.

The PPV in this study was 89% for BI-RADS 5 and 50% for BI-RADS 4 lesions. Liberman et al. found that the PPV of a BI-RADS 4 diagnosis was 23% – 34% while for BI-RADS 5 was 80% – 93%.^24^ Lazarus et al. found a PPV of 91% for BI-RADS 5 lesions.^25^ The PPV in this study is concordant with the efficacy of the BI-RADS classification system used in other studies, despite the patient’s age or breast density. Alternative BI-RADS 4 diagnoses were lymphoma, Castleman disease and pseudoangiomatous haemangioma.

A large proportion of the cancers were 2 cm – 3 cm in size in this study, although size was one of the data variables that was poorly documented in radiology reports. A case-control study of young breast cancer patients by Chung et al. found not only a median tumour size of 2.0 cm in the young group but also that tumour size was a major prognostic factor of poorer disease-free survival.^26^ Narod et al. found that cancers diagnosed at less than 2 cm has a better prognosis.^27^ It is important to diagnose breast cancers in all patients while they are still small.

Invasive ductal carcinoma was the most common histological type in patients diagnosed with breast cancer and correlates with American findings which also found IDC as the most common in all age groups.^2^ Anders et al. not only showed that ILC was the second most common histological type but also detailed that both cancers have similar manifestations.^11^ The top three histological types in the study by Eugênio et al. were IDC, then ILC and, lastly, mucinous or medullary carcinoma at similar rates,^12^ similar to this study.

This study found that 10% of patients had triple-negative breast cancer. Many studies have documented that patients under the age of 40 years have worse survival rates than their older counterparts.^25^ The triple-negative subtype is an aggressive subtype with poor survival^16^ and a worldwide breast cancer burden of 10% – 20% of invasive breast cancers.^27,28^ The majority of patients, in this study, had luminal A subtype which was also the most common finding in those younger than 40 years in a study by Zhiyang et al.^29^ Hormone receptor-positive subtypes have more treatment options available in terms of hormone-directed therapy.^16^

Using abdominal USG on the initial screening or diagnosis of a patient in a developing country is helpful in limiting the number of hospital visits for patients. A quick liver assessment can streamline patients who need further imaging. In our hospitals, any patient with lymphadenopathy or liver lesions is booked for a staging scan. Abdominal USG have also diagnosed benign lesions such as hepatic haemangiomas and gallstones.

In this study population, 3.5% (16) of patients were diagnosed with lymphoma and 30% (5) of these cases presented with bilateral breast masses. Common manifestations of breast lymphoma include enlarging mass(es), skin retraction, erythema, peau d’orange with nipple retraction and discharge.^30^ The median age for lymphoma is similar to that of breast cancer, being 60–65 years.^30^ The incidence of lymphoma in patients in Africa is not well-documented, but Naidoo et al. found that the global incidence of Hodgkin lymphoma increased because of HIV and that there was an increased incidence in the 25–49-year age group, with a mean age of 33.4 years.^31^ Unfortunately, the present study did not take the patients’ HIV status into account. Currently, there are minimal published data on the relationship between HIV and breast cancer.^32^ A South African study by Cubasch et al. details that antiretroviral treatment leads to an ‘enhanced life expectancy, shifting the distribution of cancer diagnoses towards non-AIDS-defining malignancies, including breast cancer’.^28^ A literature review found that in West Africa, HIV-positive women between 35 and 45 years were presenting with breast cancer at ages 10–15 years younger than their uninfected counterparts.^32^ The previously mentioned South African study had similar findings of ‘HIV-positive patients with breast cancer being younger than their HIV-negative counterparts’^28^ and may play a role in our study finding of increasing incidence of young breast cancers.

Studies are now researching a possible viral component to breast cancer, HIV genomes and outcomes of patients with dual diagnoses.^32^ Reddy et al. reported that the general consensus was that there was no relationship between HIV and breast cancer and that there was literature to suggest a protective role from either the virus itself or the antiretroviral treatment taken by HIV-positive patients.^33^ In sub-Saharan Africa, HIV is associated with some of the other diagnoses in this study, namely Kaposi sarcoma and lymphoma.^32^

### Study limitations

The sample for this study was a very specific at-risk population with a high referral base to the specialised breast units. The highly specific study population made cases of breast cancer much higher than if the population was more general. The reports in this study lacked information on multifocal/multicentric disease and metastases. A structured report for breast imaging could help radiologists include all the relevant information. The time interval may not depict a true increase in the number of cases and a longer time interval with the inclusion of all young patients seen may have been more conclusive.

### Future applications

The finding of breast cancer, in patients 40 years and younger, in large academic hospitals, needs further evaluation. A detailed look into risk factors, HIV burden, screening programmes and patient follow-up, is a good extension of this study. Further extension of this study to include disease progression, treatment offered, fertility status, genetic mutation and recurrence will help understand breast cancer as a complete entity in young patients. Information from all breast units should be included in the database of patients with breast cancer in South Africa, regardless of age. This database which is run currently by pathologists should include all information from involved stakeholders (nurses, clinicians, radiologists and pathologists) and be regularly updated and published.

The results of this study and future studies will help to highlight the importance of breast self-examinations, breast awareness week and breast screening services for females of all ages. Finally, healthcare practitioners need to ensure that we teach breast self-examination in all our interactions with female patients, regardless of age.

## Conclusion

Breast cancer is one of the most common cancers affecting women worldwide. This study showed that from 2016 there was an increase in the number of cases of breast cancer in patients younger than 40 years in two academic hospitals. The patient sample had similar histological and molecular subtypes as those found in international studies.

It is widely accepted around the world that breast cancer in female patients under the age of 40 years is a different disease entity and affects patients differently, in terms of aggressiveness, treatment options, recurrence rates, fertility rates and socioeconomic impact. This study has shown that breast cancer is prevalent in young patients and therefore, we need to build an efficient referral system and ensure quick, holistic care of patients. Lastly, education is key; patients younger than 40 years need to know the warning signs of breast cancer for early detection and treatment.

## References

[1] Union for International Cancer Control. Globocan 2020: New global cancer data [homepage on the Internet]. 2020 [cited 2019 May]. Available from: https://gco.iarc.fr/today/data/factsheet/populations/710-south-africa-sheets.pdf

[2] National Cancer Registry, National Institute for Communicable Diseases (NICD), National Health Laboratory Services (NHLS). Cancer statistics [homepage on the Internet]. 2009 [cited 2019 Apr]. Available from: https://www.cansa.org.za/south-african-cancer-statistics/

[3] Shah T, Guraya S. Breast cancer screening programs: Review of merits, demerits, and recent recommendations practiced across the world. J Microsc Ultrastruct. 2017;5(2):59–69. 10.1016/j.jmau.2016.10.002 30023238 PMC6025760

[4] The American Cancer Society Medical and Editorial Content Team. What is breast cancer? [homepage on the Internet]. 2021 [cited 2019 May]. Available from: https://www.cancer.org/content/dam/CRC/PDF/Public/8577.00.pdf

[5] Hamdi Y, Abdeljaoued-Tej I, Zatchi AA, et al. Cancer in Africa: The untold story. Front Oncol. 2021;11:650117. 10.3389/fonc.2021.650117 33937056 PMC8082106

[6] National Department of Health Republic of South Africa. Breast cancer control policy [homepage on the Internet]. Department of Health; 2017 [cited 2019 Apr]. Available from: https://health.gov.za/wp-content/uploads/2021/07/breast-cancer-policy.pdf

[7] Lehman CD, Lee CI, Loving VA, Portillo MS, Peacock S, Demartini WB. Accuracy and value of breast ultrasound for primary imaging evaluation of symptomatic women 30–39 years of age. Am J Roentgenol. 2012;199(5):1169–1177. 10.2214/AJR.12.8842 23096195

[8] McAree B, O’Donnell ME, Spence A, Lioe TF, McManus DT, Spence RAJ. Breast cancer in women under 40 years of age: A series of 57 cases from Northern Ireland. Breast. 2010;19(2):97–104. 10.1016/j.breast.2009.12.002 20060718

[9] Azim HA, Partridge AH. Biology of breast cancer in young women. Breast Cancer Res. 2014;16(4):427. 10.1186/s13058-014-0427-5 25436920 PMC4303229

[10] Anders CK, Johnson R, Litton J, Phillips M, Bleyer A. Breast cancer before age 40 years. Semin Oncol. 2009;36(3):237–249. 10.1053/j.seminoncol.2009.03.001 19460581 PMC2894028

[11] Anders CK, Hsu DS, Broadwater G, et al. Young age at diagnosis correlates with worse prognosis and defines a subset of breast cancers with shared patterns of gene expression. J Clin Oncol. 2008;26(20):3324–3330. 10.1200/JCO.2007.14.2471 18612148

[12] Eugênio DSG, Souza JA, Chojniak R, Bitencourt AGV, Graziano L, Marques EF. Breast cancer diagnosed before the 40 years: Imaging findings and correlation with histology and molecular subtype. Appl Cancer Res. 2017;37(1):16. 10.1186/s41241-017-0019-7

[13] Ruddy KJ, Gelber S, Tamimi RM, et al. Breast cancer presentation and diagnostic delays in young women. Cancer. 2014;120(1):20–25. 10.1002/cncr.28287 24347383

[14] Partridge AH, Hughes ME, Warner ET, et al. Subtype-dependent relationship between young age at diagnosis and breast cancer survival. J Clin Oncol. 2016;34(27):3308–3314. 10.1200/JCO.2015.65.8013 27480155

[15] Eugênio DSG, Souza JA, Chojniak R, Bitencourt AGV, Graziano L, Souza EF. Breast cancer features in women under the age of 40 years. Rev Assoc Med Bras. 2016;62(8):755–761. 10.1590/1806-9282.62.08.755 27992016

[16] Cathcart-Rake EJ, Ruddy KJ, Bleyer A, Johnson RH. Breast cancer in adolescent and young adult women under the age of 40 years. JCO Oncol Pract 2021;17(6):305–313. 10.1200/op.20.00793 33449828

[17] Johnson RH, Anders CK, Litton JK, Ruddy KJ, Bleyer A. Breast cancer in adolescents and young adults. Pediatr Blood Cancer. 2018;65(12):e27397. 10.1002/pbc.27397 30156052 PMC6192832

[18] Smilg JS. Are you dense? The implications and imaging of the dense breast. S Afr J Radiol. 2018;22(2):1356. 10.4102/sajr.v22i2.1356 PMC6837771 31754514

[19] Timmers JMH, Van Doorne-Nagtegaal HJ, Zonderland HM, et al. The breast imaging reporting and data system (BI-RADS) in the Dutch breast cancer screening programme: Its role as an assessment and stratification tool. Eur Radiol. 2012;22(8):1717–1723. 10.1007/s00330-012-2409-2 22415412 PMC3387359

[20] Cancer Research UK. Breast cancer incidence (invasive) [homepage on the Internet]. 2022 [cited 2023 Jan]. Available from: https://www.cancerresearchuk.org/about-cancer/about-our-information

[21] Dodelzon K, Starikov A, Reichman M, et al. Breast cancer in women under age 40: A decade of trend analysis at a single institution. J Clin Imaging. 2021;78:165–170. 10.1016/j.clinimag.2021.03.031 33836424

[22] Stachs A, Stubert J, Reimer T, Hartmann S. Benign breast disease in women. Dtsch Arztebl Int. 2019;116(33–34):565–573 [cited 2023 Jan]. 10.3238/arztebl.2019.0565 31554551 PMC6794703

[23] American Cancer Society. Breast cancer facts and figures [homepage on the Internet]. 2019 [cited 2020 Mar]. Available from: https://www.cancer.org/content/dam/cancer-org/research/cancer-facts-and-statistics/breast-cancer-facts-and-figures/breast-cancer-facts-and-figures-2019-2020.pdf

[24] Liberman L, Abramson AF, Squires FB, Glassman JR, Morris EA, Dershaw DD. The breast imaging reporting and data system: Positive predictive value of mammographic features and final assessment categories. Am J Roentgenol. 1998;171(1):35–40. 10.2214/ajr.171.1.9648759 9648759

[25] Lazarus E, Mainiero MB, Schepps B, Koelliker SL, Livingston LS. BI-RADS lexicon for US and mammography: Interobserver variability and positive predictive value. Radiology. 2006;239(2):385–391. 10.1148/radiol.2392042127 16569780

[26] Chung WP, Lee KT, Chen YP, et al. The prognosis of early-stage breast cancer in extremely young female patients. Medicine. 2021;100(1):E24076. 10.1097/MD.0000000000024076 33429771 PMC7793391

[27] Narod SA. Breast cancer in young women. Nat Rev Clin Oncol. 2012;9(8):460–470. 10.1038/nrclinonc.2012.102 22733233

[28] Cubasch H, Ruff P, Joffe M, et al. South African breast cancer and HIV outcomes study: Methods and baseline assessment. J Glob Oncol. 2017;3(2):114–124. 10.1200/JGO.2015.002675 28706996 PMC5493271

[29] Liu Z, Sahli Z, Wang Y, Wolff AC, Cope LM, Umbricht CB. Young age at diagnosis is associated with worse prognosis in the Luminal A breast cancer subtype: A retrospective institutional cohort study. Breast Cancer Res Treat. 2018;172(3):689–702. 10.1007/s10549-018-4950-4 30225619 PMC6786966

[30] Raj SD, Shurafa M, Shah Z, Raj KM, Fishman MDC, Dialani VM. Primary and secondary breast lymphoma: Clinical, pathologic, and multimodality imaging review. Radiographics. 2019;39(3):610–625. 10.1148/rg.2019180097 30924754

[31] Naidoo N, Abayomi A, Locketz C, Musaigwa F, Grewal R. Incidence of Hodgkin lymphoma in HIV-positive and HIV-negative patients at a tertiary hospital in South Africa (2005–2016) and comparison with other African countries. S Afr Med J. 2018;108(7):563–567. 10.7196/SAMJ.2018.v108i7.12844 30004343

[32] Grover S, Martei YM, Puri P, et al. Breast cancer and HIV in sub-Saharan Africa: A complex relationship. J Glob Oncol. 2018;2018(4):1–11. 10.1200/JGO.2016.006585 PMC6180795 30241185

[33] Reddy P, Ebrahim S, Singh B, Ramklass S, Buccimazza I. Breast cancer and HIV: A South African perspective and a critical review of the literature. S Afr J Surg. 2017;55(1):10–15. 28876552

